# Extensive experience of disease control with gefitinib and the role of prognostic markers

**DOI:** 10.1038/sj.bjc.6601476

**Published:** 2003-12-10

**Authors:** H Cortes-Funes, H Soto Parra

**Affiliations:** 1 Medical Oncology Service, Hospital Universitario 12 de Octubre, Avda Córdoba Km 5,4, Madrid 28041, Spain; 2 Istituto Clinico Humanitas, Rozzano 20089, Italy

**Keywords:** gefitinib (‘Iressa’, ZD1839), EGFR, clinical benefit, disease control, prognostic marker

## Abstract

Traditionally, the efficacy of an anticancer agent has been measured by response rate. With the development of biological molecular-targeted agents, which have a different mechanism of action from conventional agents, it may be appropriate to consider alternative criteria that reflect the positive effect of these biological agents on disease control, palliation, symptom improvement and quality of life. One such targeted agent is the orally active epidermal growth factor receptor tyrosine kinase inhibitor gefitinib (‘Iressa’, ZD1839). This article reviews the clinical experience of patients with advanced/metastatic non-small-cell lung cancer, who have received gefitinib as part of a clinical trial or through the ‘Iressa’ Expanded Access Programme. Disease-control rates of ∼50% were observed in some Expanded Access Programme series, comparable with results obtained from Phase II trials. Symptom improvement was also reported. Information that will help identify those patients most likely to respond to treatment will become increasingly important. Therefore, the possible role of prognostic markers and the relationship between epidermal growth factor receptor status and response to gefitinib has been investigated. No clear association between epidermal growth factor receptor expression and response was observed. Future studies of other biomarkers in the epidermal growth factor receptor pathway should help to identify which patients are likely to benefit most from gefitinib.

Patients with advanced/metastatic non-small-cell lung cancer (NSCLC) have terminal disease. Therefore, treatment should aim to improve palliation, quality of life and symptom improvement in addition to prolonging survival. The benefits of biologically targeted agents like gefitinib (‘Iressa’, ZD1839) are not always evident from response rates, as their mechanism of action differs from that of conventional cytotoxic chemotherapy agents. Assessments of efficacy should take into account the overall benefit to the patient, including disease control and effects on quality of life and symptoms. In addition to efficacy data from clinical trials, data are now available from the ‘Iressa’ Expanded Access Programme (EAP). Some of these data are reviewed below.

Currently, there is much interest in the role of prognostic/predictive factors and, in the future, these will become increasingly important in targeting therapy to those patients who are most likely to benefit. A range of baseline demographic factors and potential predictive biological markers are under investigation in patients receiving gefitinib, the significance of which remains to be clarified.

## CLINICAL BENEFIT WITH GEFITINIB

Unprecedented activity in NSCLC patients who have been pretreated was demonstrated in Phase II gefitinib monotherapy studies (‘Iressa’ Dose Evaluation in Advanced Lung cancer) (IDEAL 1 and 2) (Fukuoka *et al*, 2003; Kris *et al*, 2003). Response rates with gefitinib 250 mg day^−1^ were 18.4 and 11.8% in IDEAL 1 and 2, respectively. However, when disease control was considered (objective response plus stable disease), it was evident that 54.4 and 42.2% of patients in IDEAL 1 and 2, respectively, experienced clinical benefit. Furthermore, ∼40% of patients in each trial experienced improvement in disease-related symptoms, assessed using the Lung Cancer Subscale (LCS) of the Functional Assessment of Cancer Therapy-Lung (FACT-L) questionnaire (improvement rates at 250 mg day^−1^ were 40.3 and 43.1%, respectively), which was associated with a longer overall survival compared with patients without improvement (Douillard *et al*, 2002; Natale *et al*, 2002; Cella *et al*, 2003). These results emphasise that, for a targeted agent like gefitinib, valuable clinical benefit can be experienced by the patient that might not be reflected by the response rate.

Similar results have been observed in the compassionate-use setting, an example of which was presented at the American Society of Clinical Oncology annual meeting 2003 (López Martin *et al*, 2003), with updated results presented at the recent ‘Iressa’ Clinical Experience (ICE) meeting (Cortes-Funes, ICE abs). Patients were included in the ‘Iressa’ EAP if they had experienced progression of NSCLC after prior chemotherapy, were ineligible for other gefitinib studies and had no alternative treatment options. In this study, patients received treatment with oral gefitinib 250 mg day^−1^ for at least 1 month, which was the minimum time to evaluate efficacy. In addition to response rate, disease control rate (defined as objective response plus stable disease) and clinical benefit (defined in this analysis as an improvement in two principal variables, such as pain or Eastern Cooperative Oncology Group performance status, or symptomatic improvement, such as cough or haemoptysis, plus improvement in one secondary variable, such as weight) were also recorded. Data from 26 centres throughout Spain were analysed retrospectively. The four major centres were Clinica Universitaria Navarra, Hospital de Pontevedra, Hospital GTP Badalona and Hospital Universitario 12 de Octubre.

Demographic characteristics of the 113 patients from this Spanish series who fulfilled EAP criteria are given in Table 1

**Table 1:** Spanish Expanded Access Programme experience – patient demography

Patients fulfilling EAP in retrospective analysis (*n*)	113
Male/female (*n*)	89/24
Median age (range) (years)	61 (36–83)
ECOG performance status 0–1/2–3/4 (%)	73/26/1
	
*Histology (%)*	
Adenocarcinoma	41.4
Squamous-cell carcinoma	39.6
Large-cell carcinoma	15.3
Other	3.6
	
*Sites of measurable lesions (n)*	
Primary/lung	102
Nodes	44
Bone	25
Liver	15
Suprarenal	12
Central nervous system	10
Pleural	6
Adrenal	3
Other^a^	4
	
*No of sites*	
1	48
2	30
⩾3	35
	
*Previous chemotherapy (n)*	
First line	24
Second line	55
Third line	34

a Includes thoracic wall, supraclavicular, mediastinum and kidney.

EAP=Expanded Access Programme; ECOG=Eastern Cooperative Oncology Group.

**Figure 1:**
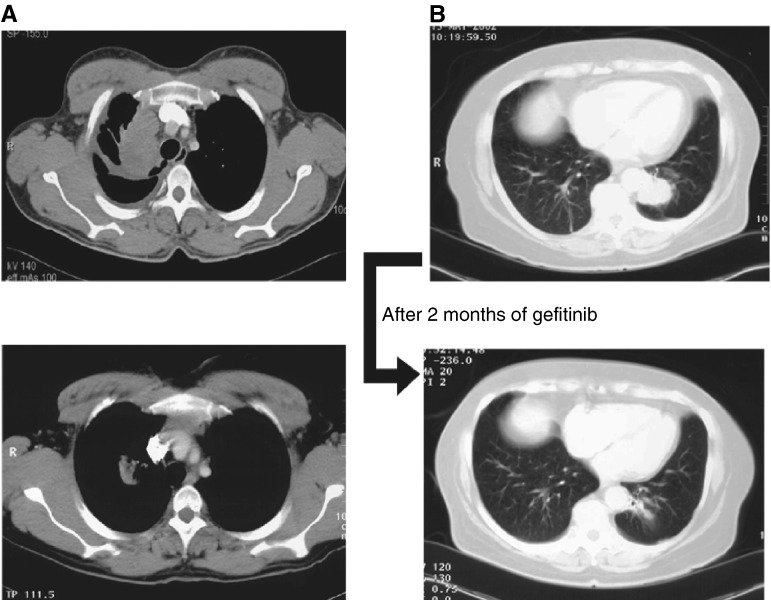
Scans showing partial response in (**A**) second- and (**B**) third-line adenocarcinoma.

. In total, 96 patients were evaluable for response, with the remaining 17 patients being evaluable only for clinical benefit. Seven patients (7.3%) had a partial response, examples of which are shown in Figures 1A and B

**Figure 2:**
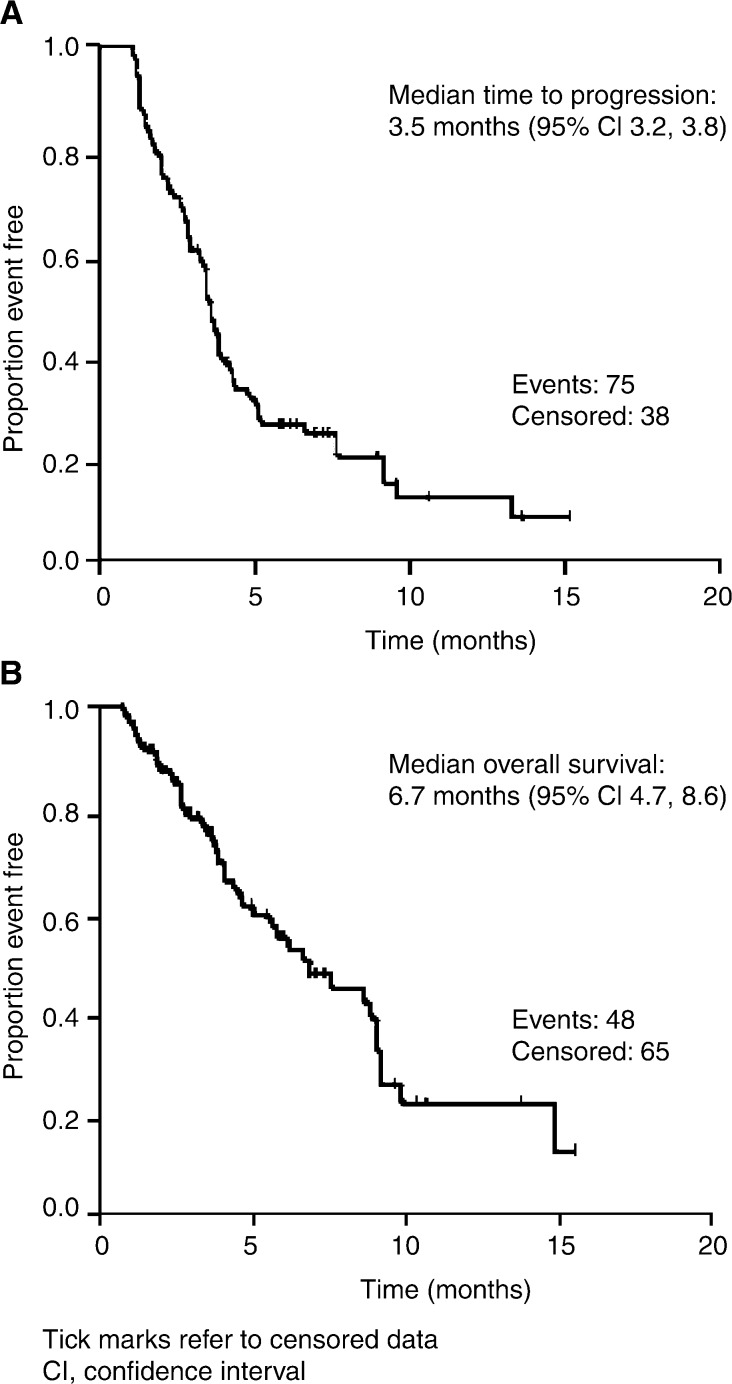
(**A**) Time to progression and (**B**) overall survival for the 113 evaluable patients from the Spanish case series.

. Disease control was experienced by 44 (45.8%) patients and disease control rates were similar, regardless of histology, stage at diagnosis or previous chemotherapy. Additionally, 23 out of 113 patients (20.4%) experienced symptom benefit (evaluated retrospectively from patient charts). Clinical benefit, as defined above, was observed in 59.2% of patients. Median time to disease progression and median overall survival were 3.5 and 6.7 months, respectively (Figures 2A and B

).

Drug-related adverse events recorded in this study were mainly grade 1/2 (Table 2

**Table 2:** Spanish Expanded Access Programme experience – most common drug-related adverse events (in ⩾5% of patients, *n*=113)

	**Grade 1/2**	**Grade 3/4**
Skin disorders, *n* (%)	48 (42.5)	4 (3.5)
Asthenia, *n* (%)	23 (20.4)	6 (5.3)
		
*Gastrointestinal disorders, n (%)*		
Diarrhoea	24 (21.2)	1 (0.9)
Anorexia	11 (9.7)	4 (3.5)
Nausea/vomiting	12 (0.6)	1 (0.9)
Neurological,^a^ *n* (%)	11 (9.7)	2 (1.8)
Alteration to central nervous and peripheral systems,^a^ *n* (%)	5 (4.4)	1 (0.9)

a Mainly disease related or related to previous chemotherapy.

). There were four withdrawals, due to drug-related adverse events (diarrhoea and fatigue in progressive disease). It can be concluded from the Spanish experience that gefitinib was well tolerated and showed antitumour activity in these pretreated patients with advanced NSCLC, with a promising rate of disease control and clinical benefit.

**Figure 3:**
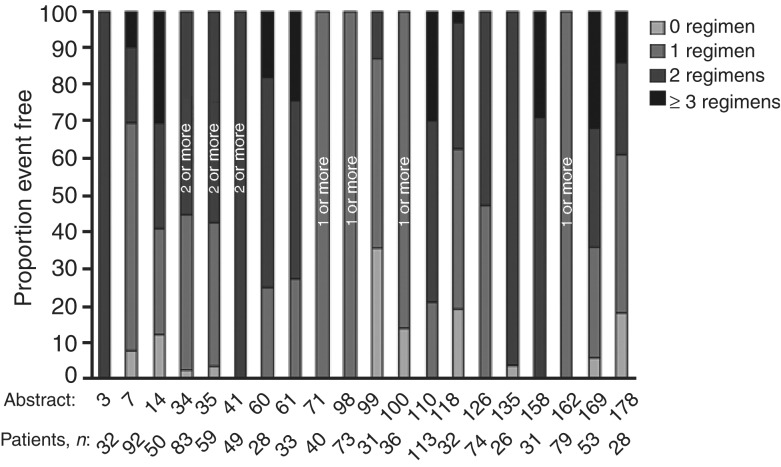
Previous chemotherapy in large case series presented at the ‘Iressa’ Clinical Experience meeting.

**Figure 4:**
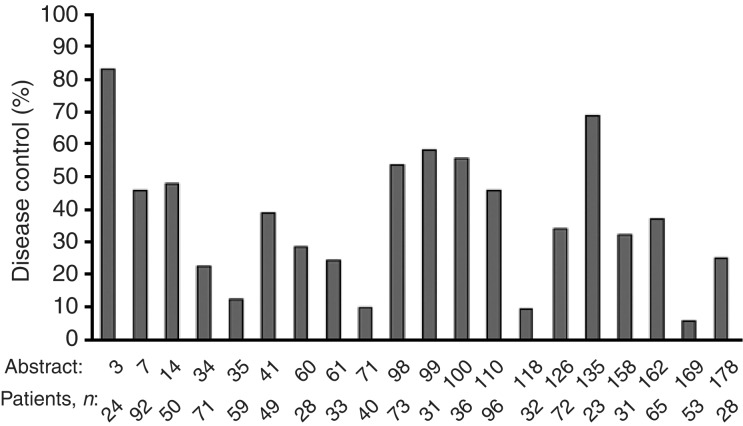
Disease control in large case series presented at the ‘Iressa’ Clinical Experience meeting.

**Figure 5:**
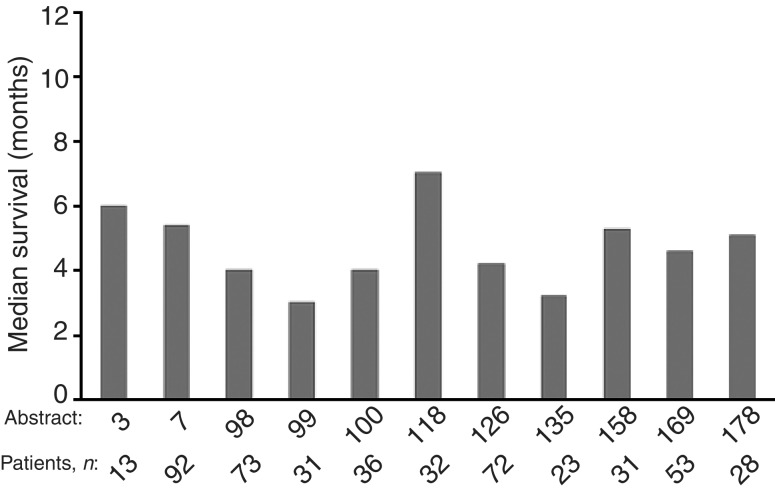
Median survival in large case series with data available at the ‘Iressa’ Clinical Experience meeting.

Efficacy data from the IDEAL trials were further supported by 20 large case series from the EAP (each with >25 patients) that were presented at the ICE meeting (Mancuso, ICE abs; Haringhuizen, ICE abs; Bendel, ICE abs; Gridelli (a and b), ICE abs; Bianco, ICE abs; de Leeuw, ICE abs; Petersen, ICE abs; Reck, ICE abs; Soto Parra (a–c), ICE abs; Cortes-Funes, ICE abs; Kowalczyk, ICE abs; Chioni, ICE abs; Katz, ICE abs; Pallis, ICE abs; de Braud, ICE abs; Razis, ICE abs; Boyer, ICE abs). Patients from these case series were commonly heavily pretreated (Figure 3

). For most case series, response rates were <10%. In contrast, disease control was experienced by many patients, with disease control rates of 5.7–83% reported across the series (Figure 4

). Some series described evidence of symptom improvement. In all, 11 of the large case series presented data on median survival (Figure 5

), which ranged from 3–7 months. The study with median survival of 7 months also reported a 1-year survival of 31% (Kowalczyk, ICE abs).

There were several presentations at the 10th World Conference on Lung Cancer (WCLC) that reported data from the compassionate-use setting (Gips *et al*, 2003; Haringhuizen *et al*, 2003; Janne *et al*, 2003; Park *et al*, 2003; Soto Parra *et al*, 2003). Response rates with gefitinib were varied, but most reports described disease control rates of >40%, comparable with data from the Phase II IDEAL trials. Furthermore, 1-year survival rates of 29% (Haringhuizen *et al*, 2003; Janne *et al*, 2003) and 48.7% (Park *et al*, 2003) were reported.

### Relationship between disease control and survival/symptom improvement

In IDEAL 2, median overall survival in the subset of patients surviving for >8 weeks was greater for patients with partial response (16.3 months) or stable disease (9.4 months) than for those with progressive disease (5.4 months) (Cella *et al*, 2003). A similar association was noted in two of the large case series from the ICE meeting that evaluated survival by disease control (Soto Parra (a and b), ICE abs). In the first, median survival was longer for patients with disease control (6 months) compared to that for all patients (4 months). In the second, median survival was 5 months for those with stable disease compared to 2.5 months for patients with progressive disease, and was 3 months overall. This study also reported a corresponding difference in 1-year survival (28% for those with stable disease *vs* 22% overall).

A correlation between symptom improvement and tumour response was observed for IDEAL 2, such that most patients with a tumour response or stable disease had symptom improvement (Cella *et al*, 2003). Four of the large case series at the ICE meeting had information on symptom-improvement rates, which ranged from 19–39% (Haringhuizen, ICE abs; Petersen, ICE abs; Kowalczyk, ICE abs; Pallis, ICE abs). In addition to an overall symptom improvement rate of 39%, Pallis *et al* noted that 83% of patients with disease control had symptom improvement.

## PROGNOSTIC MARKERS AND EPIDERMAL GROWTH FACTOR RECEPTOR STATUS

The identification of prognostic and predictive factors is important because it should help to determine which patients will benefit most from therapy. There are a number of biological markers involved in cell signalling pathways, including epidermal growth factor receptor (EGFR), p27 and Ki67, that might have potential as prognostic/predictive markers (Fu *et al*, 1999; Arteaga, 2002). Although EGFR is commonly expressed in NSCLC and other tumours, its role as a prognostic factor is not well defined and conflicting results have been reported (Nicholson *et al*, 2001). This may, in part, be due to the lack of a standard validated method for assessing EGFR expression, which makes it difficult to compare results from different studies (Arteaga, 2002). It is not known whether the level of EGFR expression is predictive of response to EGFR-targeted therapy. Recent data on the relationship between EGFR expression levels and the response to gefitinib are now available from the Phase II IDEAL trials and the EAP.

An exploratory analysis of EGFR expression in tumour biopsies has recently been performed by immunohistochemistry in the IDEAL 1 and 2 trials, in which formalin-fixed, paraffin-embedded sections were stained with the 2-18C9 clone antibody. Four levels of staining intensity were recorded: none (0), weak (1+), moderate (2+) or strong (3+). This method of visual scoring of immunohistochemical stains for EGFR by experienced observers, using a simple and objective scoring system, was shown to be highly reproducible (Janas *et al*, 2003). Patients evaluable for EGFR status plus either objective response or symptom improvement were assessed. There was no evidence for a consistent relationship between EGFR expression levels and response (Bailey *et al*, 2003). Patients with low and high levels of EGFR responded to gefitinib. There was a tendency towards a positive correlation between symptom improvement and strong (3+) EGFR membrane staining, but some patients with symptom improvement had samples without strong staining. Therefore, it would be clinically unacceptable to select patients for gefitinib treatment on the basis of strong EGFR staining.

An Italian case series from the EAP aimed to evaluate the role of EGFR detection on prediction of response or disease control in patients treated with gefitinib. Results from 50 evaluable patients were presented recently at the WCLC (Soto Parra *et al*, 2003), updated from data presented at the ICE meeting (Soto Parra (c), ICE abs). Consistent with the studies described above, response rate was 10% (one complete and four partial responses) and 50% of patients experienced disease control with a median duration of 6 months.

**Figure 6:**
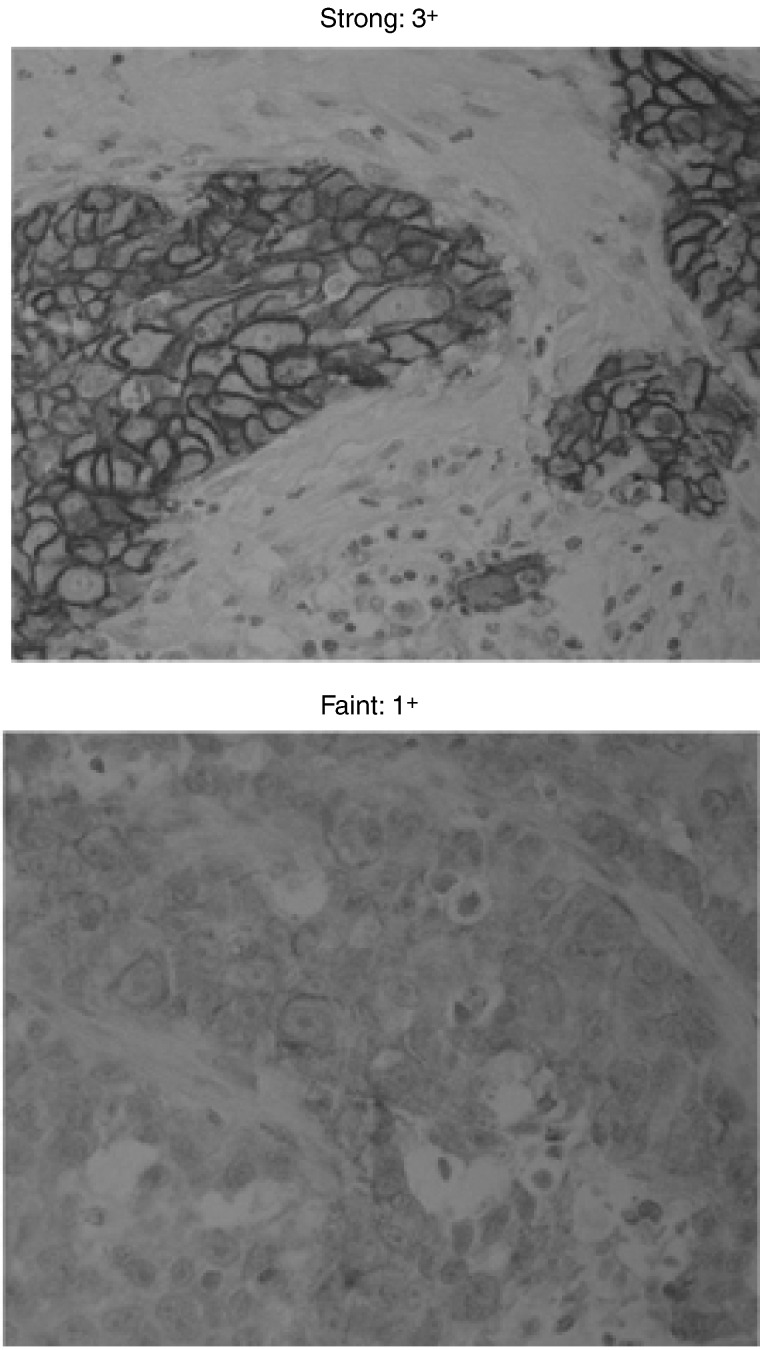
Example of staining intensity.

Immunohistochemistry was used to analyse EGFR membrane immunoreactivity, which was classified according to staining intensity (negative/faint (0/1+) or medium/strong (2+/3+)) (Figure 6

) and percentage of immunoreactive (IR) cells (negative/low expressors (0–19% IR) or high expressors (⩾20% IR)). In this exploratory analysis, there was no significant correlation between response to gefitinib and EGFR staining intensity (*P*=0.1) or between disease control and EGFR staining intensity (*P*=0.39) (Table 3

**Table 3:** Italian Expanded Access Programme experience – epidermal growth factor receptor status by response and disease control

	**Response (CR+PR)**		**Disease control (CR+PR+SD)**	
	**Yes (*n*=5)**	**No (*n*=45)**	**Yes (*n*=5)**	**No (*n*=45)**
*Staining intensity*				
2+/3+	4 (80%)	19 (42%)	10 (40%)	13 (52%)
0/1+	1 (20%)	26 (58%)	15 (60%)	12 (48%)

CR=complete response; PR=partial response; SD=stable disease.

), in agreement with the results of Bailey *et al* (2003).

In a selection of other cases from the EAP, variable results were observed: some responders were negative for EGFR (Gridelli (b), ICE abs) and others had positive staining for EGFR in ⩾20% of cells (de Braud, ICE abs). These results again suggest the lack of a clear association between EGFR status and response to gefitinib.

The IDEAL trials also provided some information on demographic prognostic factors. In IDEAL 1, prognostic factors associated with an objective response according to multivariate analysis included performance status (0/1 *vs* 2), female gender and adenocarcinoma histology (*vs* other histologies) (Fukuoka *et al*, 2003).

The Phase III trials of gefitinib in combination with standard chemotherapy in previously untreated patients with NSCLC (INTACT (‘Iressa’ NSCLC Trial Assessing Combination Treatment) 1 and 2) also included analysis of possible prognostic factors (Giaccone *et al*, 2003; Herbst *et al*, 2003). Multivariate analysis showed that performance status 2, weight loss, bone/liver metastases, squamous cell, large cell or unspecified histology were all significant for worse survival in both trials, as were male gender and brain metastases in INTACT 2. Multivariate analysis of INTACT data did not show any consistent or demonstrable effects of gefitinib in combination with chemotherapy, compared with chemotherapy alone, on known prognostic factors for survival outcome.

Another possibility for response prediction currently being evaluated is the use of a high-throughput reverse transcriptase–polymerase chain reaction assay, to determine a correlation between quantitative gene expression in the tumour and response to gefitinib monotherapy in patients with NSCLC (Natale *et al*, 2003). Of 192 genes profiled in 17 patients, expression of several genes (including STAT5A, STAT5B and *γ*-catenin) correlated with clinical response. Further studies in a larger cohort of patients are necessary to further elucidate the significance of these genes and other candidate markers of response.

## DISCUSSION

It is clear from clinical trials that patients can experience clinical benefit from gefitinib that is not necessarily reflected in the objective response rate and so other factors, such as disease control and symptom improvement, should be taken into consideration when evaluating such targeted agents. Large case series from the EAP have been presented at international congresses and at the ICE meeting, and support the idea that patients can still experience benefit despite low response rates. Some studies suggested that disease control was related to improved survival. These data indicate that gefitinib is a promising therapy for patients with advanced NSCLC, who have few treatment options.

Another important feature of targeted therapy is the potential ability to predict response to therapy. For EGFR-targeted agents such as gefitinib, it is of interest to determine any relationship between EGFR expression levels and response, with a view to being able to select for treatment those patients who are most likely to benefit. However, it appears that there is no consistent relationship between EGFR status and response to gefitinib.

There do seem to be some patterns emerging with respect to prognostic factors, with characteristics of performance status, gender and histology influencing survival, although responses were observed in all groups. In some studies, symptom improvement has been associated with improved survival (Cella *et al*, 2003).

Future studies of other biomarkers in the EGFR pathway should help identify which patients are likely to benefit most from gefitinib, and extensive investigations are underway to help clarify the mechanisms of action and resistance. Tissue collection will be performed across gefitinib trials and genomic/proteomic collaborative exploratory studies are ongoing.
